# A Data Ingestion Procedure towards a Medical Images Repository

**DOI:** 10.3390/s24154985

**Published:** 2024-08-01

**Authors:** Mauricio Solar, Victor Castañeda, Ricardo Ñanculef, Lioubov Dombrovskaia, Mauricio Araya

**Affiliations:** 1 Departamento de Informática, Universidad Tecnica Federico Santa Maria, Campus Vitacura-Santiago, Vitacura 7660251, Chile; 2 DETEM, Faculty of Medicine, Universidad de Chile, Independencia-Santiago, Santiago 8380453, Chile; vcastane@uchile.cl; 3 Departamento de Informática, Universidad Tecnica Federico Santa Maria, Campus San Joaquin-Santiago, Santiago 8940897, Chile; ricardo.nanculef@usm.cl (R.Ñ.); lioubov.dombrovskaia@usm.cl (L.D.); 4 Departamento de Informática, Universidad Tecnica Federico Santa Maria, Campus Casa Central-Valparaíso, Valparaíso 2390123, Chile; mauricio.araya@usm.cl

**Keywords:** interoperability, interoperable platform, HL7 FHIR, DICOM, data ingestion, anonymizer

## Abstract

This article presents an ingestion procedure towards an interoperable repository called ALPACS (Anonymized Local Picture Archiving and Communication System). ALPACS provides services to clinical and hospital users, who can access the repository data through an Artificial Intelligence (AI) application called PROXIMITY. This article shows the automated procedure for data ingestion from the medical imaging provider to the ALPACS repository. The data ingestion procedure was successfully applied by the data provider (Hospital Clínico de la Universidad de Chile, HCUCH) using a pseudo-anonymization algorithm at the source, thereby ensuring that the privacy of patients’ sensitive data is respected. Data transfer was carried out using international communication standards for health systems, which allows for replication of the procedure by other institutions that provide medical images. Objectives: This article aims to create a repository of 33,000 medical CT images and 33,000 diagnostic reports with international standards (HL7 HAPI FHIR, DICOM, SNOMED). This goal requires devising a data ingestion procedure that can be replicated by other provider institutions, guaranteeing data privacy by implementing a pseudo-anonymization algorithm at the source, and generating labels from annotations via NLP. Methodology: Our approach involves hybrid on-premise/cloud deployment of PACS and FHIR services, including transfer services for anonymized data to populate the repository through a structured ingestion procedure. We used NLP over the diagnostic reports to generate annotations, which were then used to train ML algorithms for content-based similar exam recovery. Outcomes: We successfully implemented ALPACS and PROXIMITY 2.0, ingesting almost 19,000 thorax CT exams to date along with their corresponding reports.

## 1. Introduction

Radiologists face the daily challenge of reviewing dozens of imaging exams at their diagnostic stations in clinical health centers. Their work consists of searching for signs or findings, which are interpreted in the particular context of each patient. In the most complex cases it is necessary to rely on various sources to determine the nature of a finding; thus, radiological impressions must be as informative as possible for the physician who requested the exam. Books, online search engines, medical atlases, and peers are the most used consultation methods for nourishing impressions. Books offer an organized and validated compilation of findings, but only provide a limited number of image examples to contrast with, which is a drawback for non-typical cases. Online search engines offer many annotated cases, but navigating and selecting results is time-consuming, limiting their use. Finally, peers can represent an up-to-date source with experience in non-typical cases, but their synchronous access is limited.

Picture Archiving and Communication Systems (PACS) correspond to computer systems for storage, review, and reporting of imaging examinations [1]. PACS offers numerous advantages compared to traditional analogical systems, even for small institutions [2,3,4,5]. These include the ability to access multiple previous images and reports on demand, as well as the opportunity for multiple physicians to collaborate from different locations using the same data. Indeed, the PACS of each center stores an extensive compendium of previous cases, with various types of presentations for which interpretation or radiological impression has been provided by a peer. While PACS has the characteristic of being automatically searchable at any time, there is a persistent problem involving searches based on annotated terms, which making it challenging to retrieve signs or imaging findings similar to the content of the consulted image. This is an obstacle for cases that need a proper differential diagnosis. Our work proposes using the potential of PACS as a source of consultation through search based on image content rather than solely on annotated keywords.

The advances in automatic medical image analysis due to computer vision and deep learning algorithms have encouraged new developments in Content-Based Medical Image Retrieval (CBMIR) Systems [6]. CBMIR is a technology used to search and retrieve medical images from a database using an image as a reference instead of keywords, tags, or explicit descriptions. CBMIR systems directly accept an image as input and return an ordered list of cases relevant to that image. This setting allows a physician to search and retrieve cases similar to a reference case without explicitly stating the input image features. Evidence from previous works suggests that CBMIR systems can help practitioners to save significant amounts of time, quickly moving from a reference image to a set of similar cases potentially containing diagnosis reports [2,7].

As discussed in [8], modern CBMIR pipelines include two major steps: feature extraction and feature indexing. Deep learning [9] is one of the most popular methods for vector image representation, and has proven particularly suitable for medical images [10,11]. On the other hand, large vector databases can be efficiently searched using quantization methods [12,13] or randomized data structures based on random projections and trees [8]. However, as already noted in previous seminal papers [14], annotations and evaluation methods are essential to obtain satisfactory CBMIR engines. As user information needs are often subjective and task-dependent, class labels used in typical image retrieval systems are often unsuitable for medical applications [8,15]. Crowdsourcing-based approaches [16] allow a wider variety of judgments to be created for training; however, the process may be expensive and time-consuming. Recently, multimodal approaches leveraging both vision and language information have shown promising results, allowing costly expert annotation to be circumvented [17], especially in medical applications, where many studies include findings and diagnosis reports [7,18,19]. Both CBIR and automatic multimodal supervision are possible thanks to the volume of medical images that are currently being digitally generated [20,21,22].

Consequently, a precondition to CBMIR is to define a procedure to ingest, access, and manage large amounts of medical data. Data ingestion is the process of loading data into a system, and represents the most critical step in any data analysis workflow [23]. According to [24], data ingestion involves importing large and varied data files from multiple sources into single storage, where they can be accessed and analyzed [25].

In general, providers of medical images and clinical data are wary of the safety and security that medical image repositories can provide. Indeed, privacy disclosures arouse widespread concern in other biomedical and scientific fields [26]. One way to guarantee the privacy of such data is to apply a pseudo-anonymization algorithm at the source; the provider delivers the pseudo-anonymized data, ensuring that any leak from the external repository avoids identification of the patients involved [27].

Another relevant requirement for a repository is the use of medical informatics standards for information exchange. HL7 has been the de facto standard in healthcare interoperability [28], with HL7 Fast Healthcare Interoperability Resources (HL7 FHIR) the latest standard created for exchange of clinical information [21]. We adopt HL7, FHIR, and DICOM (Digital Imaging and Communications in Medicine), ensuring interoperability with data providers and modern user applications.

According to [29], Artificial Intelligence (AI) techniques [30,31,32,33] such as Machine Learning (ML) [10,11,34,35] and Natural Language Processing (NLP) [36,37,38] are becoming a crucial part of future health data ingestion with the ability to deliver values not yet achieved. In our proposal, NLP is used to automatically identify and label annotations from radiologist reports [39], saving practitioners time.

The literature includes proposals such as Avicenna AI, a company that provides solutions for radiology based on AI (https://avicenna.ai/, accessed on 31 July 2024) [11,33]; Gleamer (https://www.gleamer.ai/, accessed on 31 July 2024), which provides a solution called Chestview, published in [10,30,31]; OXIPIT AI, which offers a solution that improves the diagnosis of chest X-rays with Chest Eye software (https://oxipit.ai/products/chesteye/, accessed on 31 July 2024); and Jacques et al. [32], who presented commercially available algorithms that successfully support radiologists in their tasks.

The results we have achieved are twofold: (i) ALPACS, an interoperable data repository for the health industry; and (ii) PROXIMITY, an application to search and retrieve similar exams from ALPACS based on an image query. Both of these advances can offer value to the medical diagnosis process [40].

The focus of this article is the data ingestion from a data provider such as HCUCH to the ALPACS repository. The following specific objectives are considered: (1) ingestion into ALPACS pseudo-anonymized medical image data from 10 years of examinations at HCUCH (33,000 chest CT scans, including diagnoses); (2) implementing an automated procedure for data ingestion from the HCUCH for data transfer, curation, pseudo-anonymization and indexing with as little human participation as possible; (3) transfer to ALPACS using international standards (HL7 FHIR and DICOM), with documentation to replicate the procedure with other health institutions; and (6) an evaluation of cloud tools for deploying ALPACS in a distributed and scalable way.

The contributions of this article are summarized as (i) making available a repository of 33,000 medical CT images and 33,000 diagnostic reports with international standards (HL7 HAPI FHIR, DICOM, SNOMED); (ii) designing a data ingestion procedure reproducible for other provider institutions; (iii) guaranteeing data privacy by implementing a pseudo-anonymization algorithm at the source; and (iv) generating labels from annotations via NLP.

## 2. ALPACS: Current Status

The identified actors and systems of ALPACS are divided into data providers, repository users, and key systems/actors detailed below.

### 2.1. Data Providers

These are the services of institutions where data are generated or hosted, like PACS as image storage system; HIS (Health Information Systems); Medical Responsible as health professional responsible for the PACS and HIS content; and ICT Manager as IT professional responsible for the PACS and HIS operation.

### 2.2. Repository Users

These are the interoperable applications that use the repository and potential users of these applications. Some of them are: CBMIR or KDD (Knowledge Data Discovery) system like health data mining application for content search (in our case, PROXIMITY); Front-ends as web access for exploring the repository’s data and metadata; OTS (off-the-shelf) apps, that are interoperable via DICOM and FHIR (e.g., MedDream viewer, FHIR explorer, etc.); Specific Apps designed by third parties for data mining; Medical-Clinical Users that use it to improve the diagnosis/treatment of patients in public/private services; Physician-Researcher User that use it to study diagnoses/treatments in depth to generate new knowledge; Statistical User that use it to generate statistical summaries for use in public/private policies; and Data Scientist User that use it to build new solutions.

### 2.3. Key Systems/Actors

These actors are identifiable by external systems or external actors, such as Pseudo-Anonymous PACS (the system that centralizes the images obtained by the PACS in an orderly and consistent manner); HIS Pseudo-Anonymous (the system that centralizes clinical metadata in an orderly, consistent manner and with privacy mechanisms); Data Broker and Integration system to interact with the diversity of PACS and HIS data providers; Pseudo-anonymization engine (PAE), which protects data privacy regarding individualization of patients and medical personnel; Data Trustee, the repository staff entrusted with authorization to reverse anonymization in the PAE in coordination with the medical manager; and ICT professional staff to establish transmission channels between PACS/HIS and DBIS.

The ALPACS service combines various tools that meet the interoperability requirements for a health system detailed below.

#### 2.3.1. HAPI/FHIR

HAPI/FHIR (https://hapifhir.io, accessed on 31 July 2024) is an open-source server that allows the storage of medical data with the HL7/FHIR communications and electronic health record standard. It enables clinical data to be stored in a standard and public format and provides a communication API through web queries that allow interaction with other software.

#### 2.3.2. PACS Orthanc

PACS Orthanc (https://www.orthanc-server.com, accessed on 31 July 2024) is a standard system for the storage and communication of medical images through the DICOM standard. Orthanc is an open-source PACS server that is lightweight and secure and provides a communication RESTfull API through web queries that facilitate integration with other software. We’ve analyzed alternatives such as DCM4CHEE, DICOMCloud, and OsiriX, determining that Orthanc was the best compromise between completeness (i.e., no adaptation needed), compatibility (i.e., FHIR and DICOMweb support), familiarity (i.e., Python and C++ languages), and costs (i.e., free software).

#### 2.3.3. Mirth Connect Integration Engine

Mirth is an open-source server used by several health institutions as a core for service communications and integration between software. It has implemented communications in different health standards (HL7v2, HL7/FHIR, and DICOM). We use it as an intercom between our servers, image providers, and users. Our implementation complies with (i) receiving the images from the different health institutions, delivering them to the anonymizing service and sending them to the PACS server for storage, and (ii) receiving the reports in the format that the health institution defines, and adapting it for standardized storage on the HAPI-FHIR server.

#### 2.3.4. Anonymizing Service

This service removes sensitive data from images, specifically patient data, and replaces these data with a patient ID defined by the service, which has no correlation to current patient information.

#### 2.3.5. VMs and Docker

Virtual Machines (VMs) having versatile and easily implemented services based on Docker containers allows for implementation on any server that manages containers of lightweight virtual machines (VMs) (Dockers). Docker provides the ability to generate multiple instances of a parallelized server for future client scaling. Traditional VMs are extensible and portable, allowing for the implementation of more complex services.

#### 2.3.6. CE-Net and XCeption Deep Neural Network

CE-Net is used to segment medical images. Thanks to its three components (feature encoding module, context extractor, and feature decoder module) and its pre-training, it allows good performance and access to its latent space for implementing PROXIMITY. XCeption allows image classification through a deep network architecture with pre-trained versions with over a million images from the ImageNet database. It performs well for image classification and provides access to its latent space used by PROXIMITY [34].

The validation of ALPACS and PROXIMITY were done in two stages. First, a relatively small dataset was ingested (i.e., COVID19) for testing PROXIMITY 1.0. In the second stage, a much larger dataset was ingested (HCUCH) using a structured procedure that we explain in the following sections.

### 2.4. The COVID-19 ALPACS Repository

In the ALPACS repository, medical images of patient chest X-rays and CT scans were ingested, allowing for the development of an intelligent application that seeks content-based proximity with other similar exams. ALPACS permits the development of interoperable services and the transmission, loading, processing, and pseudo-anonymizing of data. Initially, 223 GB of DICOM images from a COVID-19 database were used for technical validation. While this volume may seem sufficient, a CT scan is composed of multiple slices, making recovery of similar slices challenging to evaluate due to high intra-scan correlation. In this dataset, 42,943 DICOM files represented only a few dozen studies. The diversity and volume of data limited to COVID-19 diagnosis were low; for this reason, training of the neural networks was carried out with international and pretrained data.

Fortunately, an agreement with HCUCH allowed us to use the hospital’s chest CT data from a period of ten years (2010–2020), representing a large amount of data, to train a neural network. This dataset contains more than 33,000 chest CT scans with their corresponding diagnostic reports.

### 2.5. Empirical Evaluation of PROXIMITY 1.0

The criteria and methodologies were defined to evaluate PROXIMITY from a medical perspective, knowing its constraints in advance; the three signs that we used were condensation, nodules, and cysts. According to Fleischner (https://fleischner.memberclicks.net, accessed on 31 July 2024), the definition of condensation is a homogeneous increase in attenuation of the lung parenchyma that obscures the margins of the airway and vessels. Proximity criteria were defined to evaluate the degree of closeness of the results obtained according to the image/volume consulted. For a search for “unique condensation”, the following proximity criteria are used: Characteristics of the Findings (Number, Shape, Location, Distribution, Edges, Density) and Additional.

For each sign, a list of differential diagnoses was defined, i.e., pathologies that can be radiologically manifested with that sign, hoping that PROXIMITY will show the broadest possible variety, as this is a requirement to increase its value in differential diagnosis. For example, for “single condensation”, the following differential diagnoses were defined: Pneumonia, Organizing pneumonia, Pulmonary infarction, Adenocarcinoma, Abscess, Lymphoma, Atelectasis, Contusion, Wegener, Hemorrhage, Radiation, Sarcoidosis.

The results indicated the necessity of improving the search for the sign/“content” in PROXIMITY and expand the HCUCH Database (DB) when developing PROXIMITY 2.0. The following requirements were identified regarding images shown as close: (i) must not be from the same 3D image or study; (ii) must belong to different diagnostic impressions expressed in the study reports already processed by the tool; (iii) must not belong to the same patient consulted. The response is then filtered with the information stored in the FHIR server.

Two alternatives were designed to implement an interoperable repository service and intelligent search in daily clinical practice: first, integration in Radiological Stations, where the radiologist accesses the search service in ALPACS and receives PROXIMITY results with a single click. The second is the Web Query, where the radiologist downloads the image to a local computer and uploads it to the ALPACS query server for evaluation.

Two content similarity tests were performed using PROXIMITY. The DB is made up of exams of COVID-19 patients, consisting of twelve normal exams and twelve unique condensations from HCUCH. An image of “condensation” was chosen as the trigger for the search in ALPACS, asking PROXIMITY to choose ten similar cuts:

1. Test 1: PROXIMITY deployed two tests with condensation. The remaining eight (8) were cuts from typical cases or findings unrelated to the search. Five images corresponded to the same exam. An improvement for PROXIMITY 2.0 is the filtering of repeated images. In addition, the unfolded cuts have a different brightness and contrast than the original.
2. Test 2: PROXIMITY was specified to display exams from different patients. Three (3) images were obtained with similar findings or some relationship with the search, and seven (7) have unrelated findings, i.e. they are normal or without similarity with the search. The brightness and contrast problem remained, except for one image with adequate brightness.

### 2.6. Cloud Computing for ALPACS

In [41], the authors highlighted the need to provide health data in the cloud and presented the challenges, requirements, use cases, and best practices to create a cloud health data ingestion service. In our case, good practices require us to align with a “hybrid cloud ” strategy, where it is necessary to re-implement services on alternative servers and in the cloud as a contingency and backup. This is a way to scale up services without necessarily scaling up maintenance costs due to extensive storage usage [42,43].

Here we describe the main elements of this hybrid solution below.

#### 2.6.1. Replicating On-Premise Production Environment

A virtualization management system (i.e., oVirt) was installed on a system external to the ALPACS Data Center (AWS). The service deployment was replicated running adapted ALPACS VMs in the cloud. To enable NFS, an S3 Bucket Storage solution was allocated in AWS. MirthConnect was also replicated to ingest data via DICOM and Orthanc channels directly to the cloud. We have tested the deployment of the ALPACS base repository over AWS using untrained engineers in Health computing systems, showing that scaling up ALPACS is feasible.

#### 2.6.2. Automation of Data Transfer

The dataflow is as follows: first, images are received in Mirth Connect, which writes the image in a private NFS folder in the NAS server. Then, the anonymizer watches the folder, and when there is a change, it takes the image in the NFS folder and proceeds with the anonymization. Then, the original image is deleted or backed up (it depends on the programming steps), and the anonymized version of the image is saved in another folder. Mirth Connect watches the curated folder in which the anonymized images are stored, and when it observes changes, it sends the anonymized image to the final destination server. Depending on the programmed steps, the curated image can be deleted or backed up.

**Figure 1:**
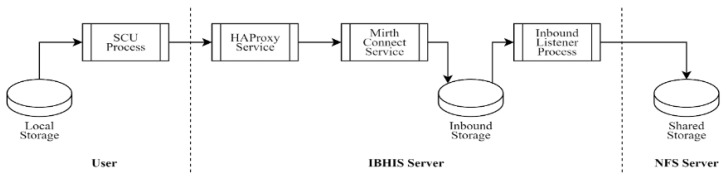
Inbound Load data flow.

**Figure 2:**
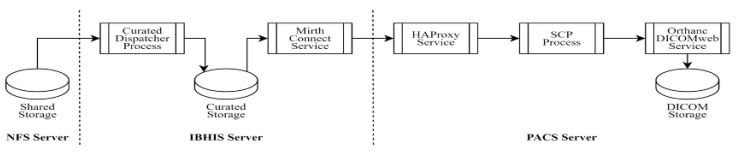
Curated load data flow.

The detailed stages of the automated data transfer process are as follows:

1. Inbound Load: The first data flow goes from the user to the NFS storage (Figure 1). It starts when the User instantiates an SCU to transmit DICOM files from its local storage to the intermediary server (IBHIS Server), which accepts TCP requests on a specific port and balances the load over TCP/DICOM channels. We have chosen HAProxy due to its popularity, but any commercial or open-source alternatives, such as NGINX, can be used. The objective is to be able to scale up transfer if needed. These files are written to memory and relocated to shared storage (S3 Bucket Storage). The IBHIS server must provide channels to transport files from a source to a destination. Here MirthConnect acts as the SCP, allowing us to cache in the inbound.
2. Curated Load: The second data flow goes from the shared storage to the PACS (Figure 2). It starts when the broker (IBHIS Server) detects that DICOM files have been uploaded to the shared storage through MirthConnect. Thus, it is ready to re-send them via TCP/DICOM. The PACS Server accepts TCP requests on a specific port and balances the load on SCU instances, which register the files in PACS (SCP) through DICOMweb. The Orthanc service stores DICOM files and provides DIMSE services and web services.
3. Archived Load: The third data stream is used to back up the archived data, which are formatted according to the PACS functions.

#### 2.6.3. Implementation of ALPACS in the Cloud

For the purposes of our implementation, Amazon Web Services (AWS) was chosen with authentication via IAM (Identity and Access Management). Deployment is done with EC2 services to instantiate and configure VMs, and S3 to provide network storage. The EC2 instances for IBHIS Server, PACS Server and FHIR Server are Debian 11 type t3.small with 2 CPUs and 2 GiB of memory. Visibility with on-premise servers is done via VPN. It was decided to migrate ALPACS services to AWS, except for perennial storage and training pipelines maintained in the ALPACS Data Center for budgetary reasons.

#### 2.6.4. HAPI/FHIR Filling

The metadata loading to the HAPI/FHIR is resumed from the metadata of the transferred DICOMs, which is not incorporated into the automatic transfer process. This is done in the curation and transformation stage.

## 3. Data Ingestion Procedure

The data ingestion procedure corresponds to the steps used to populate the ALPACS repository from a health service provider. Existing interoperability tools and data services allow this ingestion to be carried out automatically. However, effective data ingestion is part of a coordination and authorization process between institutions that cannot yet be fully automated.

### 3.1. Ingestion Roadmap

**Figure 3:**
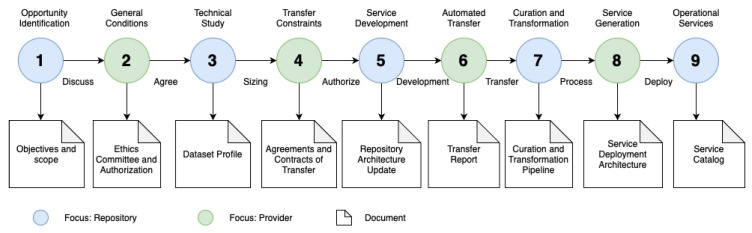
Data ingestion roadmap.

Each step of the ingestion roadmap for effective ingestion consists of a number, a title, a verb, a focus, and a target document (Figure 3). The number is a correlative that indicates the current progress of ingestion. A given step does not make it impossible to work on subsequent steps but to advance to another step, all previous steps must be completed. The title is a referential name of the step, which indicates the main challenge when executing the step. Verb proposes the “how”, i.e., describes the nature of the main activities to be carried out to meet the challenge of the step. Focus indicates which actor (repository/provider) makes the main decisions of the step, and whose execution can be unilateral, collaborative, or outsourced. The responsibility for decisions lies within the actor. The objective document is the evidence of completion of the step, where the result and progress materialize.

The roadmap is important because it shows the status of joint work and the implications of changes in the objectives and scope of data ingestion. For example, it is not unusual for new opportunities to arise as the ingestion procedure progresses, or for data that were not previously considered to later be identified as mandatory. The roadmap allows the agreed-upon ingestion to be continued while determining when to resume ingesting new data.

The details of the steps in the diagram are as follows (see Figure 3):

1. Opportunity Identification: The procedure begins with identifying common objectives between the repository and the health provider. The opportunity/benefit that the provider would obtain from providing the data to an external repository must be agreed upon, i.e., the services they would access by providing their data. This benefit can be clinical, scientific, technological, pedagogical, etc. The focus is on the repository, as it has to evaluate the feasibility of ingestion and the data mining services that can be generated from the available data. The result is a document detailing the objectives and scope of the ingestion.
2. General Conditions: Each health provider has different realities, such as policies, budgets, and governance. In this step, it is essential to agree upon the general transfer conditions that guarantee ingestion, such as necessary authorizations. The focus is on the provider, as it must approve this procedure internally. The result is a formal document supported by an ethics committee that authorizes collaboration between the provider and repository while overseeing data transfer and use.
3. Technical Study: When use of the data has been authorized, the repository must evaluate the technical feasibility of the transfer, including sizing the volume of the dataset, its formats, necessary transformations, its completeness, level of curation, the condition of the data, and the telematic networks and interoperable services used for automated transfer. The focus is on the repository, as its technical staff must complete the respective queries. The result is a document on the dataset profile that allows for analysis of the possible services to be offered and the specific data required for the transfer.
4. Transfer Constraints: Any data transfer uses health provider resources and must comply with policies, contracts, and legal constraints regarding data usage. Here, the data must be made available, i.e., resources must be assigned to carry out the transfer within the constraints mentioned above. The focus is on the provider, as its constraints shape this stage. The result is evidenced by a formal agreement/contract that enables the transfer.
5. Service Development: The repository must develop the necessary modifications to its interoperable services in order to receive/ingest the data correctly. This includes allocating the required storage, establishing communication channels, and defining data access services in a way that is interoperable with the data models. The focus is on the repository, as it must modify its software components to support the new dataset. The (internal) result is a new architecture and data model for the dataset.
6. Automated Transfer: When ingestion services are available, the provider transfers data automatically through the agreed-upon channels. The repository monitors this process, and the analysis process begins for the next curation step. The provider must assign someone responsible for restarting the transfer in case of failure. The process may need to take place over several weeks so as to not impact the provider’s networks. The focus is on the provider, who sends the data and on whom the transfer rates depend. The result corresponds to a transfer report that shows the dataset transferred to the repository.
7. Curation and Transformation: Curation and transformation corresponds to the internal process of the repository to process the data; that is, the data must be reviewed, selected, imputed, organized and transformed in order to constitute curated and coherent datasets. The process specifications must respect the conditions of Step 2, and carrying out this process with the provider is unnecessary. The focus is on the repository, as its staff and systems must perform this process semi-automatically. In addition to the curated data, the result is documentation of the modifications and adaptations of the data, which are to be delivered to the provider’s users, allowing them to use the datasets without uncertainty.
8. Service Generation: All of the services developed in Step 5 are deployed for the provider’s use and enjoyment. The focus is on the provider, as its medical departments must be integrated into the repository’s computer system, from enabling access to remote repository services to integrating the services into the provider’s RIS/PACS and HIS. The result is a service deployment architecture that integrates the repository and provider while clarifying responsibilities between the parties for maintenance, updating, and incorporation of new services.
9. Operational Services: The data access services generated by this ingestion procedure must be communicated concisely to the target audience determined in Step 2, whether they are part of the provider, the repository, or external. For this, a service catalog must be generated based on the inputs of all the previous results. This catalog must specify the form of use, its constraints, and the description of each generated dataset.

### 3.2. Implementation with HCUCH

**Figure 4:**
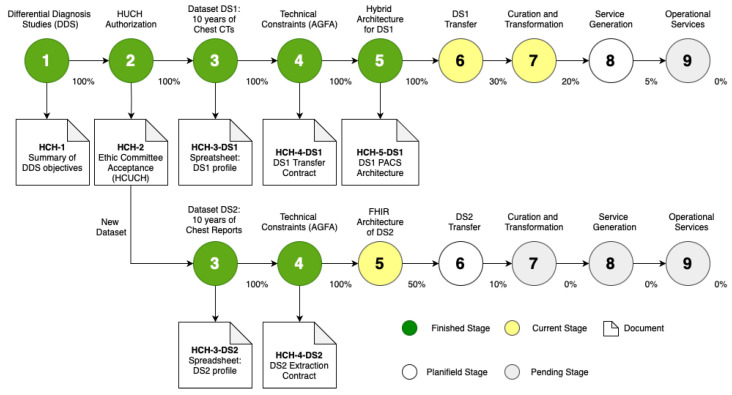
Progress status of the ingestion process in May of 2023.

Implementing the data ingestion process to the ALPACS repository of the 10-year chest CT scans obtained from the HCUCH provider is almost finalized. Here, we present a snapshot of the ingestion process one year ago (i.e., May 2023), when we were at Step 6 (Figure 4), in order to better illustrate the proposed procedure:

1. Opportunity Identification: Chest radiology was identified as a suitable domain for ALPACS objectives. A COVID-19 data-driven collaboration with HCUCH defined the goal of using ALPACS and PROXIMITY results to perform Differential Diagnostic Studies (DDS).
2. General Conditions: A project was drafted for approval by the HCUCH ethics committee detailing the medical objectives, data to be used (non-contrast chest CT scans from 2010 to 2020), and methodology to be used.
3. Technical Study: We sized the data along with the approximate volume (initially, 42,000 CTs were projected), individual volume, PACS scalability, available storage, etc. This dataset was named HCUCH DS1.
4. Transfer Constraints: The DS1 volume to be transmitted was technically approved by the HCUCH PACS administration, and negotiation with the RIS/PACS provider company, AGFA, which carried out the transmission, ensured operational continuity of the RIS/PACS while establishing ratios, low transmission rates, and an expected transmission time of several weeks.
5. Service Development: Services were adapted for the new DS1 data scale based on the ALPACS deployment. The main changes were that the provider carried out the anonymization and that a hybrid deployment was chosen, that is, while the original and curated data are hosted in ALPACS, the services usable by the provider are configured in the AWS cloud.
6. Automated Transfer: Transfer tests have been completed and the transfer has begun.
7. Curation and Transformation: The reconstructions with which the study will be carried out were agreed upon, as well as how to identify studies of the same anonymized patient. In addition, work was performed on the metadata collection systems from DICOM and radiological reports.
8. Generation of Services: Strategies were defined for deployment in the HCUCH.
9. Services Catalog: The services catalog of ALPACS.

### 3.3. New Oopportunity: Radiological Reports

During the CT ingestion procedure, in Step 5 of service development, we detected the opportunity to transfer the diagnostic reports associated with the CT data (anonymized). New advances in NLP (Natural Language Processing) based on transformers offer enough confidence to process this text in an automated way. We call this dataset HCUCH DS2.

Figure 4 shows Steps 1 and 2 completed for DS1, which are common to the DS2 dataset as well:

1. Opportunity identification: The DS2 dataset complements the CTs of DS1.
2. General Conditions: The data fit within the same authorization from the ethics committee.
3. Technical Study: The data and approximate volume were already sized (33,000 reports).
4. Transfer Constraints: DS2 transfer was coordinated via AGFA.
5. Service Development: Services for this dataset are available, and storage usage is lower.
6. Automated Transfer: A transfer is planned via interoperable protocols, although the volume of the DS2 dataset is smaller.
7. Curation and Transformation: Those of ALPACS.
8. Generation of Services: Those of ALPACS.
9. Services Catalog: Those of ALPACS.

## 4. Analysis of the Data Ingestion Procedure

In this section, we present the two main results of the data ingestion procedure: the automated transfer to ALPACS, and the development of PROXIMITY 2.0.

### 4.1. Data Transfer Statistics

Data transfer was tested with medical images in ALPACS, achieving success in this objective using different interoperable networks. This test was performed upon receipt of completeness of image datasets. Images began to be migrated from HCUCH to ALPACS servers, but not to the cloud.

We have received 8,310,064 images in the DCM file format, which correspond to 18,994 studies. Every study corresponds to a Thorax CT containing two volumes with mediastinum (approximated 200 slices) and pulmonary (approximated 100 slices) reconstruction views. Some studies also have other images of small volume reconstruction (zone of interest) and volume measurements for quantification. On average, each study has 489 images.

Every study takes 111.72 s or 1 min and 51 s (approximately 2 min) to be received and stored in the NFS folder, i.e., 228 ms per image. On average, the anonymization takes 219 ms per image, equivalent to 1 min and 47 s per study. Note that this anonymization time is when a solid-state disk is used. In the case of the NAS folder, the time is 25 min and 19 s per study, i.e., 3.1 s per image, which includes acquiring the remote image file, image processing, and writing the curated image.

The last step to send images to the final destination server takes 0.973 s and 7 min and 56 s (476 s) per study on average. Note that the time required to receive and send the images depends completely on the network and servers. In our case, the speed of the connection between the Hospital PACS and Mirth Connect is 10 Gbps, while the speed between Mirth Connect and the last server is 1 Gbps.

### 4.2. Developing Proximity 2.0

PROXIMITY was validated using eight (8) studies provided by HCUCH that were not considered in the training and were considered satisfactory preliminary validations. PROXIMITY 1.0 has a number of limitations which are surpassed by PROXIMITY 2.0:

#### 4.2.1. Study Level Recommendation

PROXIMITY 1.0 was built to work at the level of individual images. It operates on two-dimensional (2D) slices of three-dimensional (3D) volumetric studies. A study can generate between 200 and 1000 cuts. PROXIMITY 1.0 extracts each slice separately, representing a limitation on the efficiency and effectiveness of the system, as it prevents the detection of relationships between slices. PROXIMITY 2.0 was designed at the studio level using a method based on PROXIMITY 1.0 that works at the slice level. Preliminary tests on PROXIMITY 2.0 indicate that it is convenient to work with 2D slices, which reduces the parameters and computational requirements. While the base operations of PROXIMITY 2.0 are applied to 2D representations, but it works at the study level using data fusion strategies [35].

#### 4.2.2. Diversity and Novelty of Results

PROXIMITY 1.0 was trained using small international datasets and was limited to a single diagnosis. PROXIMITY 2.0 was trained on a combination of national and international datasets, an order of magnitude larger regarding images, patients, and anomalies detected. The first dataset is a sample of 100 CT images/slices from around 40 patients with segmented annotations of the image areas with three possible findings of interest: consolidation, ground glass opacification, and pleural effusion. The second dataset is a sample of 2482 CT images/slices from 120 patients with annotations about the presence/absence of SARS-CoV-2. The agreement with the HCUCH permits the use of thousands of chest CT scans, expanding and specializing the discriminative potential of PROXIMITY 2.0 in the clinical environment where it will ultimately be used.

#### 4.2.3. Automatic Annotation Extraction via NLP

PROXIMITY 1.0 was built using purely supervised transfer learning methods, which places critical limits on the ability to exploit large volumes of data due to the requirement for specialists to manually examine and annotate each study to be used in each of the findings/diagnoses of interest. This is extremely expensive in terms of specialist hours and limiting in terms of data that can be made available for training. Instead, PROXIMITY 2.0 includes NLP [36,38]. We developed AUTOLABEL, a tool that automatically extracts labels from the radiological reports that accompany each study. When AUTOLABEL detects one of the finding terms, it undergoes analysis to detect negations [39]. For this, a variant of the BERT model is used [37].

#### 4.2.4. PROXIMITY Optimization

PROXIMITY 1.0 suggested that the dimensionality of visual embeddings, such as the number of studies indexed, can significantly affect computational efficiency. PROXIMITY 2.0 includes methods to scale DBs to be larger by one or two orders of magnitude.

#### 4.2.5. New Training Mechanisms

Weakly supervised methods allow PROXIMITY to be scaled to larger datasets; however, the annotations obtained are subject to error. PROXIMITY 2.0 uses self-supervised methods that explicitly promote the consistency of the obtained predictions and representations [44]. PROXIMITY 2.0 includes training methods that avoid the extraction of pseudo-annotations, working directly on aligning visual information with the textual information available in a study.

## 5. Conclusions

In conclusion, the design of an interoperable repository that is useful for clinical users must comply with the following fundamental aspects: (i) based on international standards, such as HL7 FHIR, DICOM, and SNOMED; (ii) guarantee the privacy of stored data and pseudo-anonymized the data at the source in order to generate trust in the provider; (iii) include an automated data ingestion procedure from any provider to the repository; (iv) contain a large number of medical images and diagnostic reports; (v) include NLP technology to automatically obtain annotations from the radiological reports that accompany the studies, thereby saving time and costs in data collection; and (vi) offer a tool such as PROXIMITY for searching and retrieving images from the repository that are similar to consulted image, which offers value to medical teams (such as radiologists) in reaching a diagnose.

Please note that many drawbacks can be found in our solution, such as the costs of using cloud services, inefficiency in terms of transfer rates when using interoperable technologies, information loss due to anonymization, and lack of proper validation of the NLP techniques used for training the PROXIMITY engine on radiology reports. However, these choices were made due to real-world limitations such as budgetary constraints, standard compliance, legal and ethical requirements, and the lack of structured labels. Consequently, we believe that our ingestion procedure could help other researchers facing these real-world constraints to fill the research gaps as needed for modern AI-based solutions to reach patients.

Further work is being carried out to redesign the PROXIMITY 1.0 indexing technique using hyperbolic neural networks to generate visual embeddings. Another innovation for PROXIMITY 2.0 is the development of cross-modal training methods for intramodal image–image retrieval (im2im). This innovation derives from original contributions to knowledge that will be published in the future.

Another innovation combines automatic image processing and NLP, placing PROXIMITY 2.0 at the forefront of content-based search.

## Data Availability

Data is unavailable due to privacy restrictions.
